# Retraction Note: Chemical, electrochemical and surface studies of new metal–organic frameworks (MOF) as corrosion inhibitors for carbon steel in sulfuric acid environment

**DOI:** 10.1038/s41598-022-21190-8

**Published:** 2022-09-28

**Authors:** Abd El-Aziz S. Fouda, Safaa Eldin H. Etaiw, Gannat S. Hassan

**Affiliations:** 1 Department of Chemistry, Faculty of Science, Mansoura University, Mansoura, 35516 Egypt; 2 Department of Chemistry, Faculty of Science, Tanta University, Tanta, 31527 Egypt

Retraction of: *Scientific Reports* https://doi.org/10.1038/s41598-021-99700-3, published online 12 October 2021

The Editors have retracted this Article. After publication, concerns were raised regarding similarities between this Article and previous works from the same Authors.

Specifically:

- Figures 1, S1 and S2 appear highly similar to Figures 1, 2 and 3 in [1];
- Figure 2 appears highly similar to Figure 1 in [2];
- Figure 12 appears highly similar to Figure 14 in [3];
- Table 3 appears to overlap with Table 1 in [1], with the exception of the parameter "Formula weight".

The Authors did not respond to the requests for clarifications. The Editors therefore no longer have confidence in the presented data.

Safaa Eldin H. Etaiw agrees with the retraction and its wording. Abd El-Aziz S. Fouda did not respond to the correspondence about the retraction. The Editors were not able to obtain the current contact details for Gannat S. Hassan.
